# T174. STRUCTURAL ABNORMALITIES IN THE CINGULATE CORTEX IN ADOLESCENTS AT ULTRA-HIGH RISK WHO LATER DEVELOP PSYCHOSIS

**DOI:** 10.1093/schbul/sby016.450

**Published:** 2018-04-01

**Authors:** Adriana Fortea, Phillip van Eindhjoven, Jose Pariente, Anna Calvo, Albert Batalla, Elena de la Serna, Daniel Ilzarbe, Jordina Tor, Montserrat Dolz, Inmaculada Baeza, Gisela Sugranyes

**Affiliations:** 1 Hospital Clínic de Barcelona, Universitat de Barcelona; 2 Radboud University Medical Center, Donders Institute for Brain Cognition and Behavior, Centre for Cognitive Neuroimaging; 3 Magnetic Resonance Image Core Facility, Institut d’Investigacions Biomèdiques Agustí Pi i Sunyer (IDIBAPS); 4 Hospital Clinic of Barcelona, Centro de Investigación Biomédica en Red de Salud Mental (CIBERSAM); 5 Hospital Clinic of Barcelona; 6 Hospital Sant Joan de Déu, Centro de Investigación Biomédica en Red de Salud Mental (CIBERSAM); 7 Hospital Sant Joan de Déu, Centro de Investigación Biomédica en Red de Salud Mental (CIBERSAM); 8 Hospital Clinic of Barcelona, Centro de Investigación Biomédica en Red de Salud Mental (CIBERSAM), Institut d’Investigacions Biomèdiques Agustí Pi i Sunyer (IDIBAPS)

## Abstract

**Background:**

Identification of biomarkers of transition to psychosis in individuals at ultra-high risk (UHR) has the potential to improve future outcomes (McGorry, 2008). Structural MRI studies with UHR samples have revealed steeper rates of cortical thinning in temporal, prefrontal and cingulate cortices in individuals who later develop psychosis in both baseline and longitudinal comparisons (Fusar-Poli, 2011; Cannon, 2014). However, little is known about how onset of prodromal symptoms during adolescence impacts on changes in cortical thickness (CTH) (Ziermans, 2012).

**Methods:**

Multicentre cross-sectional case-control study, including youth aged 10–17 years, recruited from two child and adolescent mental health centres. UHR individuals were identified using the Structured Interview for Prodromal Syndromes criteria with some modifications. Healthy controls (HC) were recruited from the same geographical area. Exclusion criteria comprised personal history of psychotic symptoms, IQ<70, autism spectrum disorder, presence of neurological disorder, or antecedents of head trauma with loss of consciousness. The study was approved by the local Ethical Review Boards. All participants underwent a comprehensive socio-demographic and clinical evaluation at baseline and after 6, 12 and 18 months follow-up to identify which individuals converted to psychosis (UHR-P) and which did not (UHR-NP).

High-resolution magnetic resonance structural images were acquired at baseline on a 3Tesla and 1.5Tesla scanners. An inter-site compatibility study was conducted with healthy controls which revealed high inter-site correlation coefficients (r>.6) for CTH measures. Images were pre-processed employing automated procedures implemented in FreeSurfer 5.3.0, cortical parcellation employed the Desikan-Killiany brain atlas. Analyses: First, mean global and lobar (frontal, parietal, temporal, occipital, insula and cingulate) CTH measurements were computed. Then, within lobes showing group effects, CTH was measured for each parcellation. ANCOVA was performed to test differences between groups in SPSS 22.0, including gender, age, total intracranial volume and site as covariates. Significance was set at p<.05, corrected using the false discovery rate (FDR).

**Results:**

122 subjects were included (59 UHR-NP vs. 18 UHR-P vs. 45 HC, mean ages: 15.2 ± 1.5 vs. 15.0 ± 1.8 vs. 15.8 ± 1.5, F=1.9, p=.15; gender (%female): 61.0% vs 61.1% vs 68.9%, χ2=.76, p=.68). There were no significant differences in case-control proportion between centres: χ2=1.3, p=.25. No significant differences in global CTH in UHR-P (2.57 ± 0.13mm) relative to UHR-NP (2.56 ± 0.11mm) and HC (2.58 ± 0.09mm) were found.

There was a significant group effect on the right cingulate cortex (F=6.6, pFDR=.024): UHR-P showed lower CTH in this area relative to controls (p=.007 uncorrected). Within the right cingulate cortex, a significant group effect was found in the posterior cingulate (F=5.7, pFDR=.016) and isthmus (F=4.6, pFDR=.024), and a trend level in the caudal anterior cingulate (F=2.9, p=.057): with smaller CTH in UHR-P relative to HC in the isthmus cingulate (p=.025) and the posterior cingulate (p=.066). No significant differences were observed between UHR-P and UHR-NP groups.

**Discussion:**

UHR-P showed significant cortical thinning in several regions of the right cingulate cortex in comparison to HC, giving support to the notion that structural alterations in the cingulate cortex may be present in children and adolescents prior the onset of psychosis. Longitudinal changes in CTH have the potential to increase understanding of changes related to transition to clinical illness.

